# Comprehensive MALDI-TOF Biotyping of the Non-Redundant Harvard *Pseudomonas aeruginosa* PA14 Transposon Insertion Mutant Library

**DOI:** 10.1371/journal.pone.0117144

**Published:** 2015-02-09

**Authors:** Tonio Oumeraci, Vanessa Jensen, Steven R. Talbot, Winfried Hofmann, Markus Kostrzewa, Brigitte Schlegelberger, Nils von Neuhoff, Susanne Häussler

**Affiliations:** 1 Institute of Cell and Molecular Pathology, Hannover Medical School (MHH), Hannover, Germany; 2 Department of Molecular Bacteriology, Helmholtz Center for Infection Research, Braunschweig, Germany; 3 Institute of Molecular Bacteriology, Twincore, Center for Experimental and Clinical Infection Research, A joint venture of the Helmholtz Center for Infection Research Braunschweig and the MHH, Hannover, Germany; 4 Institute of Molecular and Cell Physiology, MHH, Hannover, Germany; 5 Bruker Daltonik GmbH, Bremen, Germany; Universitätsklinikum Hamburg-Eppendorf, GERMANY

## Abstract

**Background:**

*Pseudomonas aeruginosa* is a gram-negative bacterium that is ubiquitously present in the aerobic biosphere. As an antibiotic-resistant facultative pathogen, it is a major cause of hospital-acquired infections. Its rapid and accurate identification is crucial in clinical and therapeutic environments.

**Methods:**

In a large-scale MALDI-TOF mass spectrometry-based screen of the Harvard transposon insertion mutant library of *P. aeruginosa* strain PA14, intact-cell proteome profile spectra of 5547 PA14 transposon mutants exhibiting a plethora of different phenotypes were acquired and analyzed.

**Results:**

Of all *P. aeruginosa* PA14 mutant profiles 99.7% were correctly identified as *P. aeruginosa* with the Biotyper software on the species level. On the strain level, 99.99% of the profiles were mapped to five different individual *P. aeruginosa* Biotyper database entries. A principal component analysis-based approach was used to determine the most important discriminatory mass features between these Biotyper groups. Although technical replicas were consistently categorized to specific Biotyper groups in 94.2% of the mutant profiles, biological replicas were not, indicating that the distinct proteotypes are affected by growth conditions.

**Conclusions:**

The PA14 mutant profile collection presented here constitutes the largest coherent *P. aeruginosa* MALDI-TOF spectral dataset publicly available today. Transposon insertions in thousands of different *P. aeruginosa* genes did not affect species identification from MALDI-TOF mass spectra, clearly demonstrating the robustness of the approach. However, the assignment of the individual spectra to sub-groups proved to be non-consistent in biological replicas, indicating that the differentiation between biotyper groups in this nosocomial pathogen is unassured.

## Introduction

Although a broad range of bacterial identification systems are commercially available, microbial identification based on matrix-assisted laser desorption/ionization time-of-fight mass spectrometry (MALDI-TOF MS) is currently making its way into routine bacteriological practice at a rapid pace [1–3]. While the idea of differentiating bacteria through mass spectrometric techniques dates back to 1981 [4], biotyping by intact-cell MALDI-TOF MS was initially described for bacterial species in 1996 [5]. Today, MALDI-TOF MS biotyping provides an easy-to-use, rapid and reliable proteomic alternative to established phenotypic techniques such as the Vitek2 system [6]. Combining low sample preparation costs with high-throughput capabilities, MALDI-TOF MS biotyping is exceptionally well-suited for routine use and has huge implications for clinical applications [7–9]. Sufficiently sensitive MALDI-TOF instruments are commonly available at moderate costs, the underlying database and algorithm structures used for assigning unknown bacterial profiles to know strains however, have developed at a much slower rate [10–11]. Correct strain identification seems to be strongly influenced by the size and quality of the database spectra collection as well as by the investigated proteomic fingerprint range [12–13]. Provided a well-organized database, MALDI-TOF MS biotyping results were shown to be very robust in terms of colony-to-colony differences even if the culture conditions were not identical [14–17]. The high reproducibility could be owed to the fact that the proteomic MALDI-TOF MS fingerprint is largely based on ribosomal proteins that are constitutively expressed in the cell in very high abundance and thus not subject to the expression variability seen in phenotypic methods [18–20]. In a systematic large-scale approach, we recorded the mass spectra of the comprehensive Harvard transposon insertion mutant library of the *Pseudomonas aeruginosa* strain PA14 (http://ausubellab.mgh.harvard.edu/cgi-bin/pa14/home.cgi, accessed 04–2013) [21]. The extensive MS dataset acquired in this study provides information on the robustness of MALDI-TOF MS biotyping identification and sheds light on whether knock-out mutations in thousands of different genes impact MALDI-TOF intact-cell profiles and subsequent MALDI-TOF MS biotyping.

## Methods

### Cultivation and preparation of PA14 mutants

All 5833 transposon insertion mutants of the Harvard Medical School *P*. *aeruginosa* PA14 library were transferred onto LB agar plates in batches of 96 mutants. In total, 5547 mutants exhibited sufficient growth for further analyses. PA14 mutants were prepared on target plates (MSP 96 polished steel target, Bruker Daltonics, Bremen, Germany) by standardized smear procedure and immediately overlaid with 1μL of saturated alphacyano-4-hydroxycinnamic acid matrix solution.

### MALDI-TOF MS

Intact-cell bacterial profile spectra were acquired in duplicates using a MicroflexLT MALDI-TOF device (Bruker Daltonics). Escherichia coli DH5α bacterial test standard (Bruker Daltonics) was used for external calibration. Spectra exceeding 1000 ppm maximal peak shift were excluded from further analysis as non-recalibratable and remeasured. The full raw spectra collection as universal csv data and Bruker fid spectra including detailed acquisition parameters can be downloaded from https://www3.mh-hannover.de/proteomix/pa14/spectra (user: plos1, password: dataset).

### Data Analysis

A total number of 11094 mass spectra, consisting of two replicas of 5547 PA14 mutants, were analyzed in the range of 3000–15000 m/z. The mutant spectra were grouped into subclasses according to their unambiguous Biotyper 3.1 (Bruker Daltonics, database build 66) classification scores. The resulting *P*. *aeruginosa* subclasses were PA 19955–1 CHB, PA 8147–2 CHB, PA ATCC 27853 CHB, PA DSM 1117 DSM, and PA DSM 50071 T HAM, respectively. All 11094 raw spectra were normalized to 100 arbitrary intensity units and baseline corrected by regressing the varying baselines to a set of fixed window points (250) using a spline approximation (MATLAB 7.7.0). Class specific mean spectra were calculated for each sub-class. For peak detection, a wavelet-based MATLAB 7.7.0 peak picking algorithm (mspeaks) with a base of 2 and 10 levels as well as median-absolute-deviation for noise threshold estimation was applied on each individual spectrum [22]. Rigorous peak picking was achieved by a minimum height filter of 0.3 normalized intensity units and a minimum distance of 5 m/z between peaks [23]. Only peaks with a minimum full-width-at-half-height (FWHH) of 4 were considered valid [24].

The individual peak lists were used to generate pseudo-spectra of equal length by indexing the m/z-range for each of the spectra. In the next step spectral information was decomposed by using principal component analysis (PCA) in order to reduce and harmonize the variance between the analyzed spectra. Internal validation of the model was achieved by using all scores of the resulting PCA in linear discriminant analysis (LDA), thereby securing that the covariance matrix is positive definite and the loss of variance information is minimized [25]. In comparison to the PCA model, an approach using only the pseudo-spectra as input was also followed, allowing both model validation and the classification of external biological replicas. All classification runs were stratified by 10-fold cross-validation. The PCA approach also allowed the calculation of distances between individual spectra and clusters. The cluster centroids were used to calculate *Ward’s* distance between the five classes.

## Results

### MALDI Biotyper classification of the PA14 mutant library

The main objective of this study was to elucidate to what extent the inactivation of single genes influences MALDI-TOF MS based biotyper identification. Overall, of the 11094 mass spectra generated from the entire set of 5547 PA14 mutants, 11060 (99.7%) were correctly classified as *P*. *aeruginosa* by Biotyper 3.1 analysis (using default parameters according to standard biotyper rules (the highest scoring hit with identification score > 2.0)) as shown in Table 1. Species-misidentification and ambiguous results for replicas was exclusively due to poor spectrum quality (or contamination). A detailed table with the ten highest-scoring Biotyper database (build 66) matches for each spectrum can be downloaded from https://www3.mh-hannover.de/proteomix/pa14/bt (user: plos1, password: dataset).

**Table 1:** MALDI Biotyper 3.1 classification of the PA14 mutant library.

TaxId	Species	Spectra	Proportion (%)	Mean Score
287	*Pseudomonas aeruginosa* DSM 1117 DSM	5887	53.06	2.28
287	*Pseudomonas aeruginosa* ATCC 27853 CHB	2077	18.72	2.13
287	*Pseudomonas aeruginosa* 8147_2 CHB	1424	12.84	2.17
287	*Pseudomonas aeruginosa* DSM 50071T HAM	1291	11.64	2.08
287	*Pseudomonas aeruginosa* 19955_1 CHB	381	3.43	2.13
287	*Pseudomonas aeruginosa* A07_08_Pudu FLR	1	0.01	1.79
-	Other Pseudomonas	14	0.13	1.39
562	*Escherichia coli* DH5alpha BRL	3	0.03	2.12
470	*Acinetobacter baumannii* LMG 994 HAM	2	0.02	1.31
29378	*Staphylococcus arlettae* DSM 20673 DSM	2	0.02	1.40
32001	*Alcaligenes faecalis* ssp *faecalis* DSM 30030T HAM	1	0.01	1.42
-	Other species	11	0.10	1.44
Sum		11094	100.00	

### Revealing discriminatory proteomic traits among Biotyper groups

**Fig 1:**
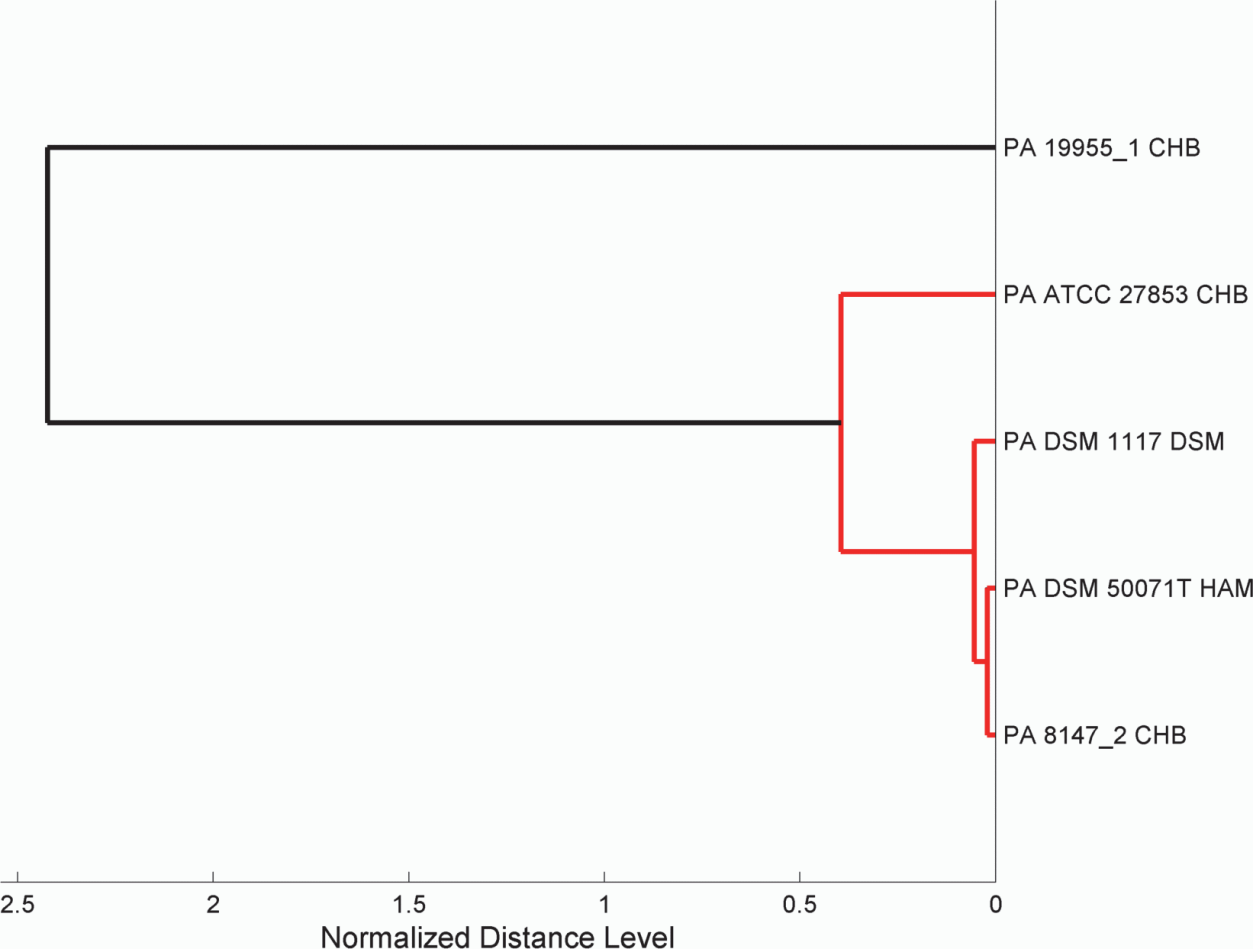
Dendrogram of the five main *P*. *aeruginosa* Biotyper database strains the PA14 mutants were mapped to. The dendrogram demonstrates that four classes are highly similar in terms of generalized spectral distances. PA 19955_1 CHB shows the largest deviation from the other four *P*. *aeruginosa* database entries.

All MALDI-TOF MS PA14 mutant profiles classified as *P*. aeruginosa were mapped to five individual Biotyper database entries (PA 19955–1 CHB, PA 8147–2 CHB, PA ATCC 27853 CHB, PA DSM 1117 DSM, and PA DSM 50071 T HAM). Fig. 1 shows a dendrogram of the five Biotyper groups based on the normalized distance level between Biotyper group-specific mean spectra. The dendrogram demonstrates that the spectra of all Biotyper groups (except PA 19955–1 CHB) are highly similar. With the aim to evaluate whether the spectral differences are caused by differences in the protein composition of the various *P*. *aeruginosa* transposon mutants we applied automatic peak detection for each Biotyper group and harmonized the variance of the spectral information by using principal component analysis (PCA) on the pooled co-variance matrix. An optimal set of discriminatory masses was selected by optimizing peak numbers and peak indices of each Biotyper group and calculating the mean correct classification rate. All classification runs were stratified by 10-fold cross-validation. Using a panel of 187 mass peaks per Biotyper group, all mutant spectra could be matched to the Biotyper groups with a mean accuracy of 99.13% (Table 2).

**Table 2:** Confusion matrix of the LDA model for classifying PA biotyper groups.

	PA 19955_1 CHB	PA 8147_2 CHB	PA ATCC 27852 CHB	PA DSM 1117 DSM	PA 50071T HAM
PA 19955_1 CHB	368	0	10	2	1
PA 8147_2 CHB	0	1403	0	6	15
PA ATCC 27852 CHB	0	2	2064	7	4
PA DSM 1117 DSM	0	11	0	5872	4
PA 50071T HAM	0	19	1	14	1257

### Robustness of the assignment of MALDI-TOF MS spectra to individual *P*. *aeruginosa* Biotyper database entries

**Fig 2:**
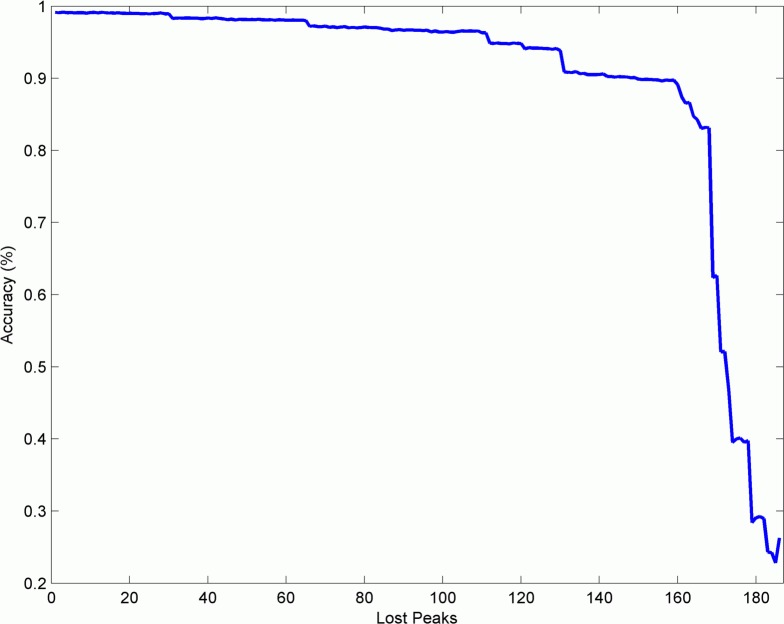
The plot shows the drop in accuracy when relevant peaks are removed. Although the classification result experiences continuous loss in accuracy when fewer peaks are included, the result is rather stable and stays above 95% until 111 peaks are removed. This indicates a highly stable peak list which can be used for mathematical subclass prediction.

To further test the potential of well-characterized proteomic panels for strain sub-classification, our MALDI-TOF MS peak-panel-based approach was evaluated in respect to its robustness. The optimized panel of 187 peaks for PA14 mutant sub-classification was highly robust in terms of Biotyper group prediction (99.13% accuracy). However, failure to detect single mass peaks for example due to inter-laboratory variability may induce a significant loss in accuracy. Therefore, we successively deleted masses from the peak list while monitoring the behavior of the accuracy using 10-fold cross-validation and discriminant analysis. The elimination of peaks continually reduced the general accuracy of the model. However, eliminating the first 111 peaks still yielded an accuracy of 95% which is an indicator for the robustness of the found model. Further peak elimination led to a more dramatic drop in accuracy. When 149 peaks were removed, the model quickly fell below 90% accuracy. Fig. 2 shows a graphic representation of this behavior which demonstrates that the combination of 187 masses forms a highly stable model for the differentiation between five of the currently 14 Biotyper groups which is also robust against the loss of multiple peaks in MALDI-TOF MS spectra. A full peaklist can be found in the supplementary material (S1 Table).

### MALDI-TOF MS spectra do not support stable classification of biological replicas

From the entire set of 5547 PA14 mutants we generated mass spectra of technical replicas of which 11060 (99.7%) were classified as *P*. *aeruginosa*. Thereby, 99.99% of the technical replicas of individual mutant profiles mapped to one of five *P*. *aeruginosa* Biotyper database entries. Table 3 shows the distribution of the technical replicas after Biotyper classification. 5.83% of the technical replicas were assigned ambiguously. We next tested whether biological replicas could be stably assigned to sub-groups. We therefore re-streaked individual *P*. *aeruginosa* transposon mutants and generated 132 MALDI-TOF-MS spectra. Unfortunately, as depicted in Table 4, 21.97% of the biological replicas were assigned unambiguously. These results clearly demonstrate that the identification of distinct clonal groups of this nosocomial pathogen is unassured.

**Table 3:** Analysis of technical replicas (MALDI Biotyper 3.1 classification).

Subgroup	No. of techn. rep. 1	No. of techn. rep. 2	Sum	Ratio 1 (%)	Ratio 2 (%)
*Pseudomonas aeruginosa* 19955_1 CHB	173	208	381	45.41	54.59
*Pseudomonas aeruginosa* 8147_2 CHB	715	709	1424	50.21	49.79
*Pseudomonas aeruginosa* ATCC 27853 CHB	1044	1033	2077	50.26	49.74
*Pseudomonas aeruginosa* DSM 1117 DSM	2963	2924	5887	50.33	49.67
*Pseudomonas aeruginosa* DSM 50071T HAM	640	651	1291	49.57	50.43
Sum	5535	5525	11060		

**Table 4:** Distribution of classified biological replicas (use of the LDA model for validating PA sub-groups).

	PA 19955_1 CHB	PA 8147_2 CHB	PA ATCC 27852 CHB	PA DSM 1117 DSM	PA 50071T HAM
PA 19955_1 CHB	103	0	2	0	1
PA 8147_2 CHB	10	0	0	0	1
PA ATCC 27852 CHB	8	0	0	0	0
PA DSM 1117 DSM	3	0	0	0	0
PA 50071T HAM	4	0	0	0	0

## Discussion

Using a fast and reproducible profiling procedure, the extraordinary robustness of the MALDI-TOF Biotyper procedure [26] was demonstrated in this study by analyzing the whole Harvard *P*. *aeruginosa* PA14 mutant library. This library comprises a total of 5833 single gene transposon insertion mutants exhibiting a plethora of different phenotypes in the genetic background of the *P*. *aeruginosa* type strain PA14. Here, this library served to generate the largest coherent microbial intact-cell MALDI-TOF MS profile spectra collection, which is now publicly available to the community (https://www.mh-hannover.de/proteomix/pa14/spectra). The simple, fast and cost effective MALDI-TOF Biotyper procedure compared very well with the Vitek2 standard routine species identification method. The obtained mass spectra correctly identified 99.7% of the studied mutants at the species level and proved to be an even more robust procedure as compared to standard biochemical testing [27].

Interestingly, all obtained MALDI-TOF MS PA14 mutant profiles that were correctly classified as *P*. aeruginosa were mapped to five individual Biotyper database entries (PA 19955–1 CHB, PA 8147–2 CHB, PA ATCC 27853 CHB, PA DSM 1117 DSM, and PA DSM 50071 T HAM). Identification of not only the bacterial species but also the affiliation to a distinct clonal group of nosocomial pathogens is expected to facilitate population dynamics studies in chronically infected patients and to improve the detection of inter-patient transmission of epidemic clones [28–29]. Only recently it was demonstrated that characteristic mass fingerprints as recorded by MALDI-TOF MS were sufficiently discriminatory to identify *Escherichia coli* subgroups as a promising tool for the optimization of infection control [30].

In this study, we evaluated whether the MALDI-TOF Biotyper could be further used for the robust identification of distinct clonal groups of the nosocomial pathogen *P*. *aeruginosa*. We found that the combination of 187 masses forms a highly stable model for the differentiation between five of the 14 currently Biotyper groups which is also robust against the loss of multiple peaks in MALDI-TOF MS spectra. However, differences between discriminatory peak positions of the clusters were only marginal and the assignment of Biotyper groups based entirely to these small shifts seemed unassured. Indeed, we found only a restricted accuracy of the classification of technical replicas and an insufficient accuracy for biological replicas.

Clearly the impact of bacterial growth conditions and/or sample preparation procedures on *P*. *aeruginosa* subtyping has to be evaluated in more detail. Thus, in conclusion although this study demonstrates that MALDI-TOF MS-based identification can routinely identify *P*. *aeruginosa* irrespective of the location of a transposon insertion to the species level with very high diagnostic accuracy, it remains to be shown that *P*. *aeruginosa* intact-cell profile spectra contain masses that prove to be suitable to accurately characterize subgroups found within the *P*. *aeruginosa* strains on the proteomic level.

## Supporting Information

S1 Table Full discriminatory peaklist of PA14 variants. (XLSX)
